# Comparison of Grip Strength Measurements by Widely Used Three Dynamometers in Outpatients Aged 60 Years and Over

**DOI:** 10.3390/jcm12134260

**Published:** 2023-06-25

**Authors:** Sumru Savas, Asli Kilavuz, Fatma Özge Kayhan Koçak, Sibel Cavdar

**Affiliations:** Division of Geriatrics, Department of Internal Medicine, School of Medicine, Ege University, Izmir 35100, Turkey; asli.kilavuz@ege.edu.tr (A.K.); drozgekayhankocak@gmail.com (F.Ö.K.K.); sibelilgun@gmail.com (S.C.)

**Keywords:** aging, muscle strength dynamometer, grip strength, sarcopenia, frailty, geriatric rehabilitation, screening

## Abstract

Grip strength (GS) is widely used in various fields such as sports, rehabilitation, and geriatrics to assess muscle strength, and to diagnose sarcopenia and frailty in older adults. There is a potential for measurement differences among different dynamometers available, and studies comparing GS measurements by variable tools have conflicting results. The two most frequently used dynamometers are the Jamar hydraulic (Jamar) and spring-type hand grip dynamometers, and Jamar has not been compared to Jamar PLUS+ Digital (Jamar+) dynamometer in older adults. So, we aimed to assess GS measurements with the Jamar as the reference standard against Jamar+, and spring-type Takei T.KK. 5401 (Takei) digital dynamometers. One hundred and ten outpatients aged >60 years were included. Inter-instrumental reliability was determined. The differences between dynamometers were evaluated by Bland–Altman plots and measurement error. The measurements with Jamar+, and Takei dynamometers were reliable and valid regarding the Jamar dynamometer. Takei and Jamar+ dynamometers overestimated GS over the Jamar dynamometer. Though the differences in the measured values might be disregarded in clinical practice, individuals defined to have low GS varied by the use of different dynamometers. Grip strength better be measured by the same dynamometer in serial measurements of older individuals.

## 1. Introduction

Muscle weakness is a component of the diagnostic criteria for major geriatric syndromes such as sarcopenia [1] and frailty [2] which are getting more and more substantial in the aging World population, thus a part of the comprehensive geriatric assessment where low muscle strength measurements predict poor outcomes such as functional decline, decreased quality of life, increased morbidity, and mortality [1,3,4,5]. Those overlapping [6,7] geriatric conditions are relevant to geriatric rehabilitation [8], and sarcopenia is closely related to physical frailty [9]. To date, numerous diagnostic definitions, and measurement methods for sarcopenia were proposed [1,10,11,12,13,14,15]. One of the operational definitions for sarcopenia is the broadly used revised European Working Group on Sarcopenia in Older People-EWGSOP (EWGSOP2) criteria that designates muscle strength as the primary parameter [1]. The choice of one of those diverse criteria and methods is generally arbitrary, and this might lead to inconsistent results in studies on sarcopenia [1,16]. As the measurement of muscle strength has become crucial for geriatric practices, reproducibility and comparability of muscle strength measurements need to be investigated.

Grip strength (GS) measurement is widely used in numerous areas such as sports, rehabilitation, and geriatrics in population-based studies, research topics, and clinical practice to assess muscle strength [3,4,11] as it is an easy and reproducible method [17,18]. Grip strength measurements by dynamometry have been reported to be reliable regarding test-retest reliability, valid, and responsive [18,19,20]. Several thresholds specific to genders, geographic regions, and/or ethnicity for normative values of GS have been defined [1,21,22,23], but there are various dynamometer alternatives for quantitative GS measurements available, and there are no device-specific thresholds. Additionally, it might be interpreted that there is no globally accepted dynamometer type or protocol for GS measurements in practice [1,24,25,26], particularly for older individuals. In addition, given the diversity of dynamometers and procedures, there exists a potential for measurement errors [27]. Grip strength measurement tools and protocols are not only variable, but they are also inadequately reported in studies [25].

Hand dynamometers for GS might be examined in four categories; hydraulic, pneumatic, mechanical (spring-type), and strain dynamometers [27,28]. The two most frequently used dynamometers are the Jamar hydraulic (referred to as Jamar hereinafter) and Smedley (spring-type) hand grip dynamometers [26]. The hydraulic Jamar dynamometer has an adjustable anatomical, and rigid handle, and an analog screen. It has high re-test reliability and precision for GS measurements [28], and it is the most cited dynamometer in the literature [27]. Though it is considered the gold standard by the American Society of Hand Therapists for measuring GS [29,30], and a validated dynamometer, some authors assume that Jamar is not up to date because of the higher weight, calibration requirement, leaking issue, and as it is not precise at lower measurements [27,30]. Additionally, for older patients with joint problems or muscle weakness, measurements with Jamar might not be accurate or easy to perform [31,32,33,34]. So, in such conditions, a pneumatic dynamometer is reported to be an option [31,32,33]. Various updated instruments have been reported to demonstrate good-to-excellent inter-instrument reliability with Jamar [30]. However, those devices are not widely used, and not available in our clinic. Furthermore, the use of the Jamar dynamometer is also limited in our region. On the other hand, it is suggested not to compare earlier data of GS by Jamar with the latest data, as GS might be higher in recent models of Jamar, and prior models might measure GS with a greater error rate [33,35]. As an alternative to Jamar, the Jamar PLUS+ Digital (referred to as Jamar+ hereinafter) has a digital display using the electronic measurement principle [30]. It is lighter than Jamar with minor differences [30]. Takei T.K.K. 5401, a spring-type dynamometer also with a digital screen presents measurements to the nearest 0.1 kg that enables operator interpretation limited, unlike Jamar. But Takei is similar to Jamar in terms of weight, and adjustable handles. Spring-type dynamometers are widely used in Asian countries [28]. Most importantly, this device is widely used in the majority of healthcare settings in our region for practice and research purposes. We could define one study comparing the measurements of the Jamar+ and Jamar dynamometer that is performed among younger adults with a lack of inter-instrument reliability [36]. To the best of our knowledge, the Jamar dynamometer has not been studied in comparison to the Jamar+ dynamometer in older adults. However, the studies investigating Jamar and other hydraulic dynamometers in comparison with Smedley dynamometers among older individuals and young to middle-aged adults have shown conflicting results [17,26,28,37,38,39,40].

So, though many comparisons with several brands, and models (digital-analog) or hydraulic and spring-type dynamometers have been carried out, comparison of GS measurements and outcomes of different dynamometers is problematic and the agreement between different hand-held dynamometers needs to be further investigated. As present variations in the features of dynamometers might affect the validity and reliability of GS measurements [26], we aimed to examine GS measurements by Jamar+ and Takei 5401 digital dynamometers with reference to the Jamar dynamometer in older outpatients in this study.

## 2. Methods

This is a cross-sectional study carried out at the Geriatrics and Internal Medicine Outpatient Clinics of Ege University Medical Faculty between May and October 2019. The participants were recruited consecutively, and the patients aged 60 years and over who gave informed consent were included in the study. Socio-demographic data, present comorbidities, anthropometric measurements, and assessments of nutritional, functional, and cognitive status by Mini Nutritional Assessment Short Form (MNA-SF) [41,42], Katz Activities of Daily Living (ADLs) [43,44], and Mini-Mental State Examination (MMSE) [45,46] were evaluated. Muscle strength was assessed by hand grip dynamometers as GS measurement. Patients who did not give informed consent, aged <60 years, with sensory deficits, finger/hand amputations, and active arthritis as well as hemiplegic/quadriplegic patients, and those with a recent (last three months) operation in upper extremities, and an acute infection/symptoms were excluded from the study. Participants with MMSE score < 24, poor nutritional status (MNA-SF score < 12), and functional dysfunction (Katz score < 5) were also excluded.

### 2.1. Measures

#### 2.1.1. Grip Strength Measurements

The GS was measured by the three hand dynamometers; the Jamar dynamometer (Performance Health Supply, Cedarburg, WI, USA), the Jamar+ dynamometer (Performance Health Supply, Cedarburg, WI, USA), and Takei T.K.K. 5401 digital dynamometer (Takei Scientific Instruments Co. Ltd., Tokyo, Japan). Both Jamar and Jamar+ dynamometers were provided through the funding of Ege University Higher Education Institutions Scientific Research & Development Project. Takei digital dynamometer was already in use in our department. Calibration of the Jamar dynamometer was performed according to the protocols set by the manufacturer. Before GS measurements, the researchers adjusted the grip size of each dynamometer according to the manufacturers’ instructions if needed. All of the measurements were performed in a silent room and around a.m. from 09.30 h to 12.30 by two trained researchers (A.K. and F.Ö.K.K.). Before starting the procedures, the participants were given verbal guidance to squeeze the dynamometers to ensure a maximum value. Then the participants squeezed the dynamometer, as hard as possible, for three seconds duration [47]. A total of nine GS measurements were performed for each patient by the dominant hand, three measurements per dynamometer with 60 s pause intervals between the trials. The participants rested for two minutes between measurements with different dynamometers. Measurements of GS were performed in an alternating sequence, in the first patient starting with the Jamar+ dynamometer, then Takei digital dynamometer, and lastly Jamar dynamometer; in the consecutive patients starting with the second dynamometer (i.e., Takei dynamometer), and then the next dynamometers in each consecutive patient. The maximum value of three GS measurements was selected for further analysis for each dynamometer. We applied the Southampton protocol for both Jamar+ and Jamar dynamometers. Participants sat in a chair holding the dynamometer with the dominant hand as the forearms rested on the arms of the chair, wrist over the end of the arm in a neutral position [27]. Dynamometers were supported at the bottom by the researchers. The procedure recommended by the National Health and Nutrition Examination Survey (NHANES) was applied to the Takei dynamometer [48]. Participants stood upright with their feet standing hip width, the shoulder neutrally positioned, each arm at the side, and the elbow extended [48].

#### 2.1.2. Anthropometric Measurements

The hand circumference (HC) (cm) was evaluated as the perimeter of the middle section of the hand at the two major transverse palmar creases. Hand length (HL) (cm) was measured by the tip of the middle finger to the midline of the distal wrist crease [49].

#### 2.1.3. Other Measures

The MMSE is a cognitive screening test with a threshold set at 24 defining ‘normal’ cognitive function [45]. The MNA-SF consists of six questions, and defines “normal nutritional status”, “at risk of malnutrition”, and “malnutrition” [41]. The performance of patients on ADLs is considered fully independent or fully dependent according to six functions [43].

### 2.2. Statistical Analysis

Normality was assessed by Shapiro-Wilk’s test, skewness, and kurtosis. The quantitative variables with a distribution consistent with the normal distribution were presented as the mean and standard deviation (SD) values. For those that differ significantly from the normal distribution, the median and interquartile range values of the data were given. Categorical variables were given as frequencies and percentages. Possible differences in general characteristics of male and female participants were tested with a *t*-test or Mann Whitney-U, and χ^2^-test whereas correlations for the GS measurements performed by different dynamometers and hand anthropometrics were analyzed by Pearson’s or Spearman correlation analysis (correlation coefficients deemed if >0.80 very strong, 0.60 to 0.79 strong, 0.40 to 0.59 moderate, 0.20 to 0.39 weak, lower than 0.19 very weak) where available [50].

To compare GS measurements by the three dynamometers, repeated measures of ANOVA and Bonferroni correction were performed. Inter-instrumental reliability of the Jamar dynamometer with the Takei and Jamar+ dynamometers was determined through intraclass correlation coefficient (ICC) analyses. ICC was performed based on a two-way random-effects model. ICC values are usually evaluated as poor, moderate, good, and excellent reliability, if <0.50, 0.50–0.75, 0.75–0.90, and >0.90, respectively. The differences between the measurements of dynamometers were evaluated by systematic bias expressed as the mean differences between the tools through Bland–Altman plots and measurement error. The normal distribution of the differences was verified. The 95% limits of agreement were defined as 1.96 standard deviations of the differences [28,51]. Standard error of measurement (SEM) and minimal detectable change (MDC) were computed using the formulations: “SEM = SD × √(1 − ICC); MDC = 1.96 × √2 × SEM [SEM% = (SEM/mean) × 100%”, and “MDC% = (MDC/mean) × 100%” (mean: average GS of dynamometers; SD: standard deviation of the differences)] [28,52,53]. Values lower than 10% or 15% for SEM% [28,54], and <30% for MDC% are suggested to show acceptable reliability [28].

There are approximately 8,000,000 individuals over the age of 65 in Turkey. To calculate the sample size, a power analysis based on ICC analysis was performed [55]. When the concordance between the 3 methods is 0.80 and above, the lowest limit of acceptability is 0.70, and at least 101 patients must be recruited to reject the H0 hypothesis at 80% power and 0.05 alpha level. However, it is a fact that there may be losses in measurement. For this reason, when the dropout rate is 10%, a minimum of 113 people should be taken. All analyses were conducted using SPSS (Statistical Package for Social Sciences) (version 25.0; SPSS, Inc, IBM Corp, Armonk, New York, NY, USA). *p* values < 0.05 were reported as statistically significant.

## 3. Results

### Participants and Clinical Characteristics

Patients aged <60 years (*n* = 102), with missing data (*n* = 38), finger/hand amputations (*n* = 2), active arthritis (*n* = 12), sensory deficits (*n* = 47), acute infection/symptoms (*n* = 92), and those who did not give informed consent (*n* = 37) were excluded from the study. After the application of the tests; participants with MMSE scores <24 (*n* = 57), poor nutritional status (*n* = 31), and functional dysfunction were also excluded (*n* = 26) (all, *n* = 444). Finally, 110 older subjects aged 60–90 years (mean 69.4 ± 6.3 years) were recruited (56.4% females). Of all, 97.3% of the study group was right-handed. The characteristics of the patients are shown in Table 1. The mean GS value of each dynamometer was significantly higher in males than in females (all *p* < 0.01). The average GS by Jamar dynamometer was lower than both Jamar+ and Takei dynamometers in the whole group (*p* = 0.004). The mean GS measurements by the Jamar dynamometer were lower than only measurements with the Takei dynamometer for women (*p* = 0.002) whereas mean values for both Jamar, and Takei dynamometers were lower than the Jamar+ dynamometer for men (*p* = 0.022) (Table 1). The mean scores of ADLs, MNA-SF, and MMSE were; 5.9 ± 0.07, 13.1 ± 0.44, and 28.5 ± 1.28, respectively.

The results of the correlational analyses between mean GS measurements by the Jamar and both Takei, and Jamar+ dynamometers are presented in Table S1a in the Online Supplement File S1: Additional Tables. Correlations between hand grip measurements of the three dynamometers and measures of the hand are presented in Table S1b in the Online Supplement File S1: Additional Tables. The overall ICC value for Jamar and Jamar+ dynamometers was deemed excellent in the total group, and good both for females and males (all *p* < 0.001) indicating a good-to-excellent inter-instrument consistency between the devices. The ICC values between the Jamar and Takei dynamometers were good in the whole group of participants, and for both genders (all *p* < 0.001). The ICC results are shown in Table 2.

In our study, all the SEM values were lower than 10%, and all MDC values were lower than <30%. Those values reflect acceptable reliability between the dynamometers. The SEM% was between 3.60% and 6.33%, and values for MDC% were from 9.99 to 17.55%. The highest, but still acceptable values were for females between the Jamar hydraulic versus Jamar+ and Takei dynamometers, in descending order (Table 2). A Bland-Altman analysis was run for the agreements of the GS measurements by the Jamar hydraulic versus Takei and Jamar+ dynamometers where variability did not increase with the mean values. Bland–Altman plots showing the differences in Jamar dynamometer measurements with Takei and Jamar+ measurements against their means are presented in Figures S1–S3 in the Online Supplement File S2: Bland–Altman Plots. There were negative mean differences regarding the total group, females and males. Mean values were close to zero, and the distribution was deemed uniform. In the total group according to the Bland-Altman plot, the Takei dynamometer overestimated GS measurements by 0.85 kg (bias: −0.85, 95% CI −1.47 to −2.22) whereas the difference was 0.98 (bias: −0.98, 95% confidence interval (CI) −1.62 to −0.34) kg for Jamar+ dynamometer over Jamar dynamometer (Figure S1a,b). Takei dynamometer and Jamar+ dynamometer measurements overestimated GS over Jamar dynamometer by 1.42 kg (bias: −1.42, 95% CI −2.16 to −0.68) and 0.76 kg (bias: 0.76, 95% CI −1.6 to 0.08) in women (Figure S2a,b), and 0.11 kg (bias: −0.11, 95% CI −1.18 to 2.97) and 1.27 kg (bias: −1.27, 95% CI −2.28 to −0.25) in men, respectively (Figure S3a,b). Linear regression analyses were also performed for the data in the whole group, female and male participants regarding the Jamar hydraulic versus Takei dynamometer and Jamar+ dynamometers reflecting no proportional bias (*p* = 0.079, *p* = 0.301, *p* = 0.945 and *p* = 0.458, *p* = 0.790, *p* = 0.887, respectively).

Low GS prevalences according to the EWGSOP2 thresholds [1] by the Jamar, Jamar+, and Takei dynamometers were 6.4%, 3.6%, 4.5 in all participants, 4.8%, 0%, 0% in women, 8.3%, 8.3%, 10.4% in men, respectively.

## 4. Discussion

Though hand GS is a widely used measurement method for muscle strength evaluation, there are multiple dynamometer types, models, brands, and protocols available. So, measurement errors or differences are likely, not limited to dynamometer-dependent factors, and the interchangeability of dynamometers is questionable. Thus, we evaluated GS measurements by the Jamar hydraulic dynamometer as a reference standard against spring-type Takei and Jamar+ dynamometers in older outpatients in this study. To the best of our knowledge, this is the first study comparing the GS measurements with Jamar and Jamar+ in older adults. Overall, in all participants, GS measured by the Jamar was lowest, values by Jamar+ in males were highest whereas the values significantly varied among females by Takei and Jamar, lower in the letter. The ICCs between the dynamometers were good to excellent indicating inter-instrumental good relative reliability. Both SEM% and MDC% values reflected acceptable reliability between the dynamometers. Takei and Jamar+ dynamometers overestimated GS over the Jamar dynamometer in females, males, and the total group, with no proportional bias noted. Low GS prevalence varied significantly by the use of different dynamometers. Hand size did not seem to have a substantial impact on GS measurements, particularly in men, but there was a variable gender effect.

In the only study we could find in the literature, addressing the validity and reliability of Jamar+ compared to Jamar, inter-instrument reliability between the tools was poor-to-moderate, with Jamar hydraulic overestimating 10% higher than Jamar+ in a small sample of 40 adults [36]. In a recent short communication utilizing the unpublished data of a mixed-aged population (aged 45–74 years), the authors reported that low GS highly varied with hydraulic, spring-gauge or electronic devices [56]. On the contrary, in our study on outpatients aged 60 years and over, we showed relative reliability, and absolute reliability between Jamar+ and Jamar whereas Jamar+ overestimated GS by clinically neglectable measurement results. In the review by Lee and Gong, the authors reported that GS measurements tend to be higher in new models of Jamar and its variants [33] because of the friction of the handles. Both Jamar dynamometers were provided for the project in our study, so, investigating and reproducing those data are essential. We could find only a few studies investigating Jamar dynamometers in comparison with spring-type dynamometers among older adults with conflicting results [26,28,37,38]. In the only study by Benton et al. on the Takei dynamometer, the authors reported excellent reliability, but poor agreement and lack of validity between the Jamar and the Smedley spring (Takei) hand grip dynamometers in 67 community-dwelling older adults. Grip strength measured with the Jamar was greater than the Smedley dynamometer while differences between dynamometers were considerably lower in women and old-old subjects (>75 years). The authors concluded that standardization and thresholds specific to dynamometers are needed [26]. This study was the only study based on the Takei dynamometer like in our study. However, the item number of the dynamometer was not provided. Likewise, in a small sample of older residents (*n* = 55) of a retirement home and social day care center, Guerra and Amaral 2009 evaluated the reliability of Jamar, Smedlay (specified as Smedlay in the article), Eisenhut, and Sammons Preston Rolyan Bulb, reporting significant differences between the dynamometers, though results of Smedlay were close to Jamar [37]. Moreover, in 467 community-dwelling older adults aged 69 to 89 years, Kim et al. indicated that they compared Jamar and spring-type Smedley (YD-100) dynamometers, and they found statistically significant differences, though there was an excellent correlation between the two dynamometers. When using the Smedley dynamometer, weakness prevalence was higher [38]. On the contrary to those studies; in a recent study by Huang et al., the authors compared the Jamar dynamometer to another spring-type dynamometer; the CAMRY EH101 dynamometer in 1064 healthy community-dwelling adults aged over 50 years, and reported that CAMRY EH101 was valid and reliable for Jamar dynamometer (systematic bias underestimated by CAMRY was 0.5 kg, and 0.6 kg in males, and females) [28]. In our study, the Takei dynamometer was valid and reliable for the Jamar dynamometer, but the Takei dynamometer overestimated GS over the Jamar dynamometer. Grip strength measurements by Takei were significantly higher than the Jamar dynamometer in women in the present study. Likewise, Guerra et al. showed that there was a variable gender effect on the differences between the dynamometers, depending on the dynamometer used [37]. Overall, in all participants, GS measured by the Jamar was lowest than both of the dynamometers in our study, contrary to most of the articles published reporting otherwise in older, mixed, and young to middle-aged populations [17,37,49]. Recently, Abe et al. showed that young to middle-aged adults with relatively higher GS values provided higher measurements by hydraulic (Baseline) than the Smedley (Takei 5401) dynamometer whereas this difference did not exist at lower GS measurements or depending on hand size [39]. Accordingly, correlations between hand GS measurements and HC or HL were weak to moderate, varying according to gender in our study. Consistent with our results, the digital dynamometer (Takei 5401) measured higher values than the hydraulic (Baseline) dynamometer with a satisfactory agreement in the study by Yu et al. among young and middle-aged adults [40]. They also reported that GS values differed with the same dynamometer, but different protocols. Though the Jamar hydraulic is generally taken as a reference standard against other devices, it is reported that there might be a potential for significant reader error for the Jamar hydraulic as the dial shows 2 kg increments [27,30]. On the other hand, Hogrel reported that during the measurement process, at the inertial movement of the needle of the Jamar dynamometer, it jumps slightly higher than the actual value which might tend to overestimate the GS value [30,49].

On the other hand, though, intra-instrument reliability and validity of diverse digital (Takei 5401), and analog (Takei 5001) models of Takei dynamometers were high in the study by Cadenas-Sanchez et al., the investigators suggested that GS should be measured with the same instrument and model for serial assessments for the same individual as inter-instrument reliability might add a certain amount of error [57]. Herein, low GS prevalence varied from zero to 10.4% according to EWGSOP2 thresholds depending on gender, and the dynamometer type used, in our study. Those prevalences might vary by the use of local thresholds, as well.

As we investigated the inter-instrumental reliability, but not the test-retest reliability, this is a limitation of the study and necessitates further investigation. Inter-rater reliability is not available in the present study. We did not entirely discuss the Jamar+ dynamometer and Takei dynamometer, as the aim of the study was to compare those instruments against the reference standard Jamar. Nevertheless, there is no other study than the present research in the literature comparing Jamar+ against a digital spring-type dynamometer in older adults.

## 5. Conclusions

The reproducibility and comparability of GS measurements by different brands, types, and models of dynamometers is a critical issue for older individuals as present few studies vary in terms of sample sizes, settings, protocols, and the characteristics of the participants. The measurements with Jamar+, and Takei dynamometers were reliable and valid regarding the Jamar dynamometer in the present study. Though the differences in the measured values might be disregarded in clinical practice, GS better be measured by the same dynamometer in serial measurements of individuals, considering the impact of gender, as well. Individuals defined to have low GS, sarcopenia, or frailty have the potential to vary by the use of different dynamometers which may affect related intervention and rehabilitation. Further studies comparing GS measurements in geriatric individuals are essential taking into consideration variable characteristics of the instruments, gender, frailty, and comorbidities as well as the protocols in order to standardize the GS measurements and to create consensus on the methodology, even instrument-specific thresholds.

## Figures and Tables

**Table 1:** The Characteristics of the participants.

Variables		All (*n* = 110)	Females (*n* = 62)	Males (*n* = 48)
Age, years		69.4 ± 6.3	68.0 ± 5.8 *	71.2 ± 6.5 *
Gender, *n* (%)	Females	62 (56.4)	62 (100)	-
	Males	48 (43.6)	-	48 (100)
Higher education, *n* (%)	Yes	31 (28.2)	16 (25.8)	15 (31.3)
Living alone, *n* (%)	Yes	12 (10.9)	10 (16.1)	2 (4.2)
Comorbid conditions, *n* (%)	Diabetes Mellitus	37 (33.6)	21 (33.9)	16 (33.3)
	Hypertension	60 (54.5)	32 (51.6)	28 (58.3)
	Coronary artery disease	22 (20)	9 (14.5)	13 (27)
	Other diseases	75 (68.2)	43 (69.3)	32 (66.7)
Measures of the hand (cm)	Circumference	21.2 ± 1.8	20.3 ± 1.1 **	22.4 ± 1.8 **
	Length	18.4 ± 1.1	17.9 ± 1 **	18.9 ± 1.1 **
Grip strength (kg)	Jamar dynamometer _1_	28.5 ± 7.7 ^†^	23.9 ± 5.0 ** ^‡^	34.3 ± 6.5 ** ^§^
	Takei dynamometer _2_	29.3 ± 7.1 ^†^	25.4 ± 4.6 ** ^‡^	34.4 ± 6.6 ** ^§^
	Jamar+ dynamometer _3_	29.5 ± 7.9 ^†^	24.7 ± 5.1 **	35.6 ± 6.6 ** ^§^

Numerical variables are expressed as mean (SD). Other diseases: Malignancy, chronic pulmonary-liver disease, thyroid disease. Jamar+; The Jamar PLUS+ Digital dynamometer. * Significance at *p* < 0.01 level between genders. ** Significance at *p* < 0.001 level between genders. ^†^ Significance at *p* < 0.01 level between the three dynamometers in the whole group (1 < 2,3). ^‡^ Significance at *p* < 0.01 level between the three dynamometers among females (1 < 2). ^§^ Significance at *p* < 0.05 level between the three dynamometers among males (1,2 < 3). _1_: The measurements with the Jamar dynamometer, _2_: The measurements with the Takei dynamometer, _3_: The measurements with the Jamar+ dynamometer.

**Table 2:** Intraclass correlation coefficient analyses, standard error of measurement, and minimal detectable change values for grip strength measured by the three dynamometers.

	ICC ^1,2^ (95% CI)	ICC ^1,3^ (95% CI)	SEM (%)	MDC (%)
All (*n* = 110)	0.899 [0.856–0.930] *	0.905 [0.865–0.934] *	3.66 ^1,2^ 3.60 ^1,3^	10.14 ^1,2^ 9.99 ^1,3^
Females (*n* = 62)	0.819 [0.716–0.887] *	0.784 [0.666–0.864] *	5.01 ^1,2^ 6.33 ^1,3^	13.89 ^1,2^ 17.55 ^1,3^
Males (*n* = 48)	0.839 [0.730–0.907] *	0.858 [0.760–0.918] *	4.33 ^1,2^ 3.76 ^1,3^	12.00 ^1,2^ 10.43 ^1,3^

ICC; intraclass correlation coefficient, SEM; standard error of measurement, MDC; minimal detectable change. ^1^ Jamar dynamometer, ^2^ Takei dynamometer, ^3^ Jamar+ dynamometer. * Significance at *p* < 0.001 level.

## Data Availability

The data presented in this study are available on request from the corresponding author. The data are not publicly available due to ethical statements.

## Supplementary Materials

The supporting information can be downloaded at: https://www.mdpi.com/article/10.3390/jcm12134260/s1, File S1: Additional Tables, and File S2: Bland-Altman Plots.
