# Supplementary material for: Comparison of Grip Strength Measurements by Widely Used Three Dynamometers in Outpatients Aged 60 Years and Over
Source: J Clin Med. 2023 Jun 25;12(13):4260. doi: 10.3390/jcm12134260 (PMC10342845; doi:10.3390/jcm12134260)
Supplement: Supplementary file 1 [file jcm-12-04260-s001.zip › Online Supplement S1 Additional Tables.pdf]

Online Supplement S1: Additional Tables (Tables S1a, and S1b)

Table S1a. Correlations between grip strength measurements of Jamar dynamometer and grip strength measurements of Takei and Jamar+ dynamometers.

	**All** (n=110)	**Females** (n=62)	**Males** (n=48)
Jamar vs. Takei	r=0.901<br>p<0.001	r=0.821<br>p<0.001	r=0.839<br>p<0.001
Jamar vs. Jamar+	r=0.906<br>p<0.001	r=0.785<br>p<0.001	r=0.858<br>p<0.001

Jamar+; The Jamar PLUS+ Digital dynamometer.

Table S1b. Correlations between grip strength measurements of Jamar, Jamar+, and Takei dynamometers and measures of the hand: hand circumference and hand length.

		**All** (n=110)	**Females** (n=62)	**Males** (n=48)
Jamar dynamometer	Jamar vs. HC	r=0.594<br>p<0.001	NS	NS
	Jamar vs. HL	r=0.501<br>p<0.001	r=0.334<br>p=0.008	NS
Jamar+ dynamometer	Jamar+ vs. HC	R=0.585<br>p<0.001	r=0.266<br>p=0.037	NS
	Jamar+ vs. HL	r=0.432<br>p<0.001	NS	NS
Takei dynamometer	Takei vs. HC	r=0.499<br>p<0.001	NS	NS
	Takei vs. HL	r=0.415<br>p<0.001	NS	NS

HC; hand circumference, HL; hand length, NS; not significant, Jamar+; The Jamar PLUS+ Digital dynamometer.
