# Supplementary material for: Comparison of Grip Strength Measurements by Widely Used Three Dynamometers in Outpatients Aged 60 Years and Over
Source: J Clin Med. 2023 Jun 25;12(13):4260. doi: 10.3390/jcm12134260 (PMC10342845; doi:10.3390/jcm12134260)
Supplement: Supplementary file 1 [file jcm-12-04260-s001.zip › Online Supplement S2 Bland¿CAltman Plots.pdf]

Online Supplement S2: Bland–Altman Plots (Figures S1, S2, and S3)

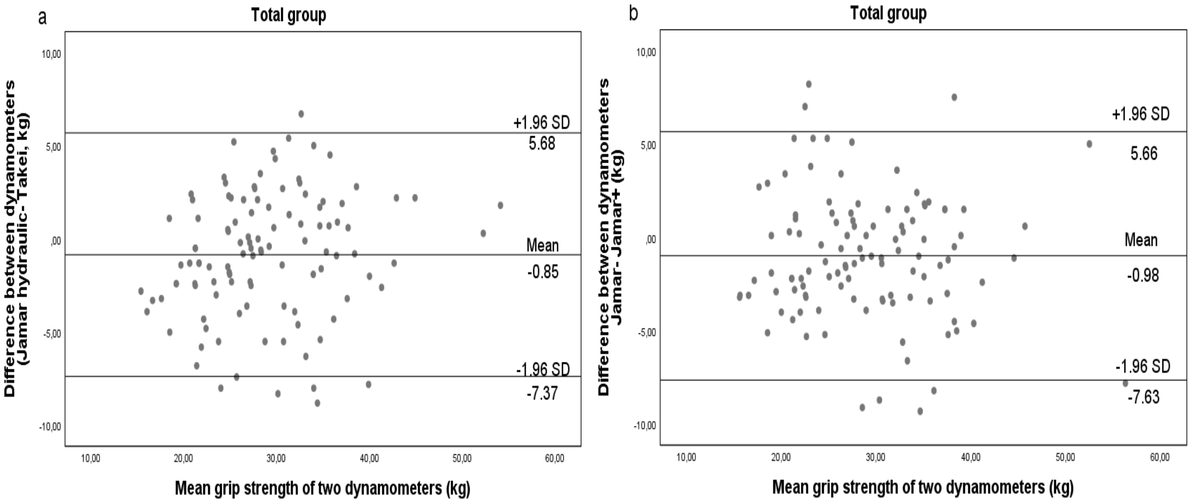

Figure S1. Bland–Altman plots showing the differences between **a**) Jamar and Takei measurements, and **b**) Jamar and Jamar+ grip strength measurements against their means in the total group. Jamar+; The Jamar PLUS+ Digital dynamometer.

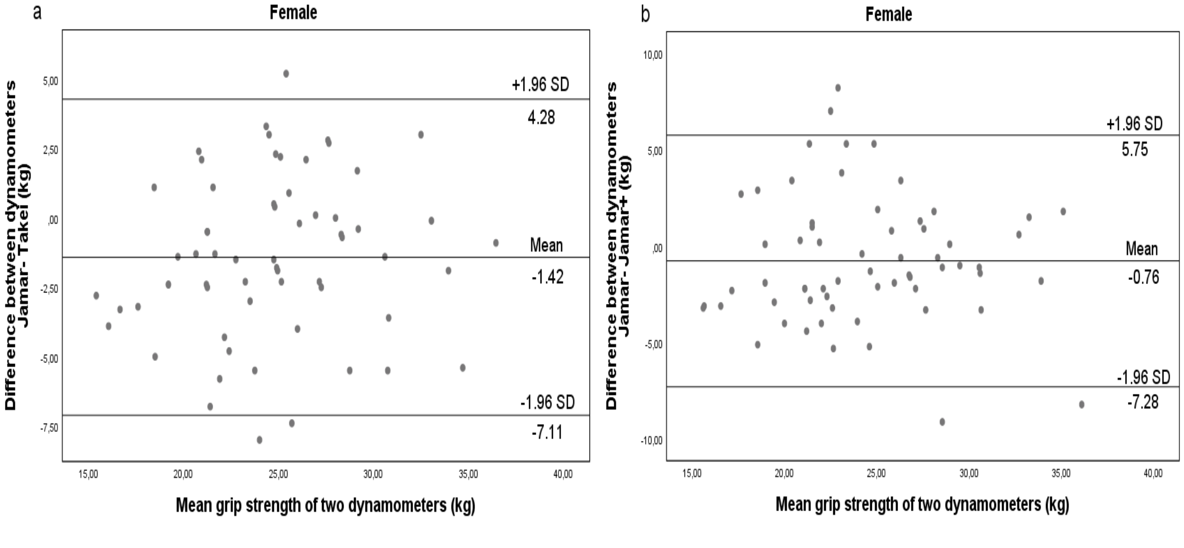

Figure S2. Bland–Altman plots showing the differences between **a**) Jamar and Takei measurements, and **b**) Jamar and Jamar+ grip strength measurements against their means in females. Jamar+; The Jamar PLUS+ Digital dynamometer.

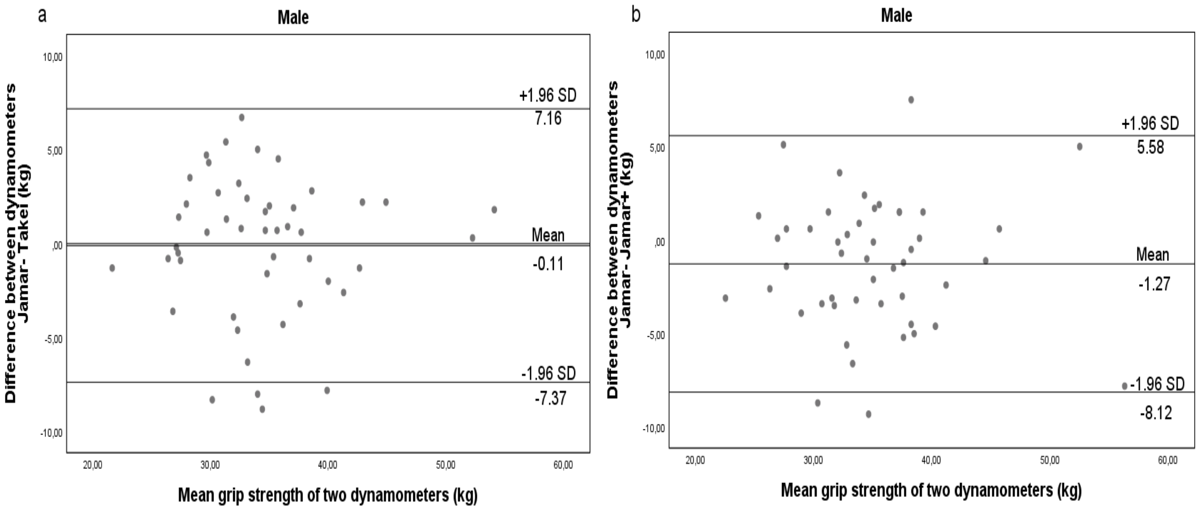

Figure S3. Bland–Altman plots showing the differences between **a**) Jamar and Takei measurements, and **b**) Jamar and Jamar+ grip strength measurements against their means in males. Jamar+; The Jamar PLUS+ Digital dynamometer.
